# Sufentanil Vs Fentanyl for Fast-Track Cardiac Anaesthesia

**Published:** 2009-08

**Authors:** C M Deshpande, S N Mohite, Prashant Kamdi

**Affiliations:** 1,2 Professor, Department of Anaesthesiology, T N Medical College & B Y L Nair Hospital, Mumbai; 3 P.G. Student, Department of Anaesthesiology, T N Medical College & B Y L Nair Hospital, Mumbai

**Keywords:** Fast Track Cardiac Anaesthesia (FTCA), Sufentanil, Fentanyl

## Abstract

**Summary:**

A perioperative anaesthetic management that aims to facilitate tracheal extubation of patients within 1-6 hrs after cardiac surgery is called “fast-track”. Main advantage of “fast-track” method is better usage of medical resources and lowering hospital costs without increasing morbidity and mortality of the patients. Standard fast-track protocols contain short acting anaesthetic agents, smaller incisions and decreased pump times without hypothermia. In this study we compared two short acting opioid drugs, fentanyl versus Sufentanil when used as a part of the balanced anaesthesia technique for fast track in cardiac surgery patients & evaluated the time taken for extubation, haemodynamic stability, analgesia requirements & incidence of awareness. The results from the study show that both agents provide good haemodynamic stability and postoperative analgesia. Although Sufentanil provides earlier extubation, both agents reduce the ICU stay equally. In conclusion both agents can be used effectively for fasttrack cardiac anaesthesia.

## Introduction

Opioids have been an integral part ofcardiac anaesthesia due to their cardiostable properties. Prolonged mechanical ventilation as a consequence of high dose opioid anaesthesia was an essential part of postoperative care in cardiac surgery during its developing years.

A perioperative anaesthetic management that aims to facilitate tracheal extubation of patients within 1-6 hrs after cardiac surgery is called ‘fast-track“^1^,^2^ (FTCA). Main advantage of “fast-track” method is better usage of medical resources and lowering hospital costs without increasing morbidity and mortality of the patients. Safety and effectiveness of fast-track versus slow-track cardiac anaesthesia is proved by many studies.^3^‐^5^ An effective fast track cardiac anaesthesia program requires appropriate selection of suitable patients, a low dose opioid anesthetic technique, early tracheal extubation, a short stay in the ICU, and coordinated perioperative care.^6^,^7^. In this study we compared the time to extubation, haemodynamic stability and postoperative analgesia when two short acting opioid drugs, fentanylor sufentanil were used as a part of the anaesthesia technique for fast trackin cardiac surgery patients. We also compared incidence of awareness associated with FTCA.

## Methods

After obtaining approval from hospital academic and ethics committee and written, informed valid consent, 100 patients between the age of 15-50 years, undergoing elective open-heart surgery for valvular and simple congenital heart disease were enrolled in this randomized, prospective, double blinded study. The study excluded patients with left ventricular ejection fraction (LVEF) <20%, severe pulmonary hypertension (PH), severe COPD, renal insufficiency, severe liver disease, history of seizure or stroke, history of allergy to propofol, patients in whom cardiopulmonary bypass (CPB) time >2 hrs and pregnant patients.

All patients underwent thorough preoperative evaluation and investigations. All the cardiac medications of the patient were continued until the morning of surgery. After arrival to the operating room, patients were administered oxygen (O_2_) by nasal prongs and monitoring of ECG (5 lead) with automated ST segment analysis and pulse oximetry was initiated (Intellivue MP 40, Philips Medical Systems, Germany). Under local anaesthesia and aseptic precautions, a 16-G intravenous cannula was inserted in the dorsum of right hand, a 20-G intra-arterial cannula was introduced into the left radial artery for monitoring of the arterial pressure and obtaining arterial blood for analysis and right internal jugularvein cannulation was done with appropriate size triple lumen cannula for CVP monitoring.

Patients were randomly divided into two groups of 50 patients each. Sufentanil group (S) received 0.5μg.kg^-1^ of sufentanil while Fentanyl group (F) received 3μg.kg^-1^ of fentanyl as part of induction. All patients were induced with IV midazolam 0.05mg.kg^-1^, a sleep dose of thiopentone sodium and IV vecuronium 0.1mg.kg^-1^ to facilitate endotracheal intubation. Patients were mechanically ventilated with tidal volume of 10 ml.kg^-1^ and respiratory rate of 10-12 / min using Pennon ventilator. Anasogastric tube and nasal temperature probe were introduced. Diclofenac suppository was inserted. Anaesthesia was maintained using oxygen, nitrous oxide, isoflurane (end tidal concentration 0.8-1%) and intermittent doses of vecuronium, and midazolam (1-5mg) before and after cardiopulmonary by pass with goal to maintain stable haemodtnarmics. On cardiopulmonary bypass, an infusion of propofol was started at the rate of 4-5 mg.kg^-1^.hr^-1^ for maintenance of anaesthesia. Additional doses of opioid drug were given at the following steps of intense stimulus-at sternotomy, just before going ‘ON’ CPB, coming ‘OFF’ CPB and as & when required as per the discretion of consultant anaesthetist. Patients from Sufentanil group received 0.1 μg.kg^-1^ of Sufentanil while patients from Fentanyl group received lμg.kg^-l^ of fentanyl as additional dose. Total amount of sufentanil, fentanyl and midazolam administered during entire procedure was restricted to 1 μg.kg^-1^, 6 μg.kg^-1^ and 5 mg respectively.

Inspired and expired gas concentration of O_2_, carbon dioxide (C0_2_) and isoflurane were measured using anaesthetic gas monitoring system (anaesthesia gas monitor, Intellivue MP 40, Philips Medical Systems, Germany). Haemodynamic parameters were maintained within 20% of the basal values with small boluses of IV nitro-glycerine/ sodium nitroprusside or IV ephedrine / phenylephrine and small boluses of IV metoprolol/ esmolol or atropine / glycopyrolate as required. Filling pressures and fluid balance was maintained using lactated Ringers solution, 6%hydroxy-ethyl starch (HAES-steril, Fresenius Kabi) and blood and blood products as necessary. All patients were given infusion of 5% glucose with 10 units of insulin and 20 mEq of potassium for myocardial protection. All the cardiac surgeries were done using normothermic cardiopulmonary bypass.

If needed, infusions of dopamine/ isoprenaline/ adrenaline were used as inotrope while coming off by-pass to maintain hemodynamics as per choice of an-aesthetist and surgeon. Chest wound and chest tube insertion sites were infiltrated with 0.25% bupivacaine, in all patients.

At the end of surgery, patients were reversed and extubated on table if

- Awake & alert
- Hemodynamically stable or minimal inotropic support
- Good tidal volume with a respiratory rate of 10-24 breaths /min and good cough reflex.

Patients were mechanically ventilated when

- Deeply sedated patient
- Unstable hemodynamics or high dose inotropic support

The mode of ventilation in all patients was as follows

- Synchronised intermittent mandatory ventilation (SIMV) + Pressure support ventilation(PSV) (10 cm H_2_O) + Continuous positive airway pressure(CPAP) (3–5 cmH_2_O)
- Tidal volume(VT) 10 ml/kg & Respiratory rate(RR) 10 breaths /min
- To keep PaCO2 < 40 mmHg & pH 7.35–7.45

Patients who were deeply sedated did not require additional sedatives or relaxants. Muscle relaxant was given only to patients with unstable hemodynamics or heavy inotropic support to reduce work of breathing till haemodynarnically stable.

These ventilated patients were weaned by standard protocol when hemodynamically stable and were extubated with or without reversal depending on clinical signs of residual neuro -muscular blockade. Patients requiring prolonged ventilation due to surgical complications such as excessive drains, need for reexploration were excluded from the study. ‘Ventilator time’ was defined as time from arrival in ICU to extubation. Prolonged ventilation was defined as continued mechanical ventilation till next day morning or for more than 12 hours. All patients were given IV ondansetron 0.8 mg.kg^-1^ before extubation.

All patients were monitored in ICU postoperatively every 15 min for first hour and then every half an hour for 6 hours. Patients were asked to rate his/her pain (0-10) using Visual Analogue Score 30 minutes after extubation and at next morning. IV tramadol was given (lmg.kg^-1^) as rescue analgesia. In awake and extubated patients trtmadol was given if VAS> 4cm or when patient demanded an analgesic. In mechanically ventilated patients, tramadol was given on clinical evidence of pain e.g. sweating, tachycardia, and hypertension.

Time of first dose of tramadol was noted and the number of doses of tramadol between arrival in ICU and next morning were also noted. Patients were also asked for awareness during surgery, one hour after extubation and at next morning. Each patient was asked a standard set of questions. 1. What is the last thing you remember before surgery? 2. What is the next thing you remember? 3. Can you remember anything in between these two periods? 4. Did you have any dreams in between these two periods?

Sample size was calculated from previous study^8^ on the basis of the anticipated difference in mean ventilation time between the two groups. Assuming Type I error of 5% and Type II error of 20% (Power 80%), a 30% reduction was considered as clinically significant i.e. to detect difference of 120 minutes with standard deviation of 202 minutes. This required a sample size of 45 patients in each group. We used 50 patients in each group.

The data obtained in this study was analyzed using either unpaired ‘t’ test or Pearson Chi-Square test according to different variables. A *p* value of less than 0.05 was considered significant.

## Results

A total of 100 patients were included in this prospective, randomized double blind study with 50 patients in each group. The two groups were comparable with regard to the demographic, preoperative and intraoperative data (Table 1,2)

**Table 1:** Statistical analysis and comparison in mean of Demographic parameters, Baseline Vital parameters, Diagnosis, CPB duration & Cross Clamp time

Parameter	Sufentanil Gp			Fentanyl Gp		*p* value	Test
			Mean+/- SD				
Age (Yrs)	27.86+/-10.587			30.88+/-10.499		0.155	‘t’ test
Weight (Kg)	46.08+/-8.351			43.40+/-9.777		0.146	
Sex Distribution	M	F		M	F	0.422	Pearson chi square
	29	21		25	25		
Diagnosis*							
Valve D/Cong.HD	41/9			41/9		0.474	Pearson chi square
Preoperative Pulse(bpm)	86.00+/-14.651			85.20+/-17.543		0.805	‘t’ test
Preoperative SBP (mm of Hg)	120.02+/-14.460			120.14+/-13.830		0.966	‘t’ test
Preoperative DBP (mm of Hg)	72.12+/-9.888			73.30+/-8.577		0.525	‘t’ test
Preoperative CVP (cm of H2O)	2.26+/-1.782			2.20+/-1.927		0.872	‘t’ test
Bypass time (min)	75.5+/-17.44			77.3+/-17.56		0.608	‘t’ test
Cross clamptime (min)	39.86+/-9.78			41.35+/-8.66		0.422	‘t’ test

* - Valvular D- Valvular Disease: Included Mitral stenosis/regurgitation, Aortic stenosis/ regurgitation or both for Valve Replacements, Cong HD- Congenital Heart Disease: Included Atrial septal defects, Ventricular septal defects and Congenital Pulmonary stenosis for repair

**Table 2:** Statistical analysis of distribution of inotropic support between the two groups

		IS * group Crosstabulation					p'value
				Group		Total	Pearson Chi-Square
Inotropic Support		SUFENTANIL			FENTANYL		
dopa 10mcg/kg	n		0		2	2	
	% within group		0%		4.0%	2.0%	0.352
dopa 8mcg/kg	n		2		1	3	
	% within group		4.0%		2.0%	3.0%	
dopa 5mcg/kg	n		1		1	2	
	% within group		2.0%		2.0%	2.0%	
dopa 4mcg/kg	n		2		7	9	
	% within group		4.0%		14.0%	9.0%	
dopa 3mcg/kg	n		1		1	2	
	% within group		2.0%		2.0%	2.0%	
nil	n		44		38	82	
	% within group		88.0%		76.0%	82.0%	
Total	n		50		50	100	
	% within group		100.0%		100.0%	100.0%	

All the patients from both groups maintained stable haernodynamics throughout the surgery and there was no statistical difference in the vital parameters between the two groups in the prebypass and postbypass period. There was no statistically significant difference in the number of patients needing inotropic support as well as the amount of inotropic support needed between the two groups in postbypass period.

The VAS score after extubation was 0.54 ± 1.417 cm in Sufentanil group and 0.32 ± 1.115 mm in Fentanyl group. The VAS score on the next day morning was 0.46 ± 0.734 cm and 0.42 ± 0.642 cm in Sufentanil and Fentanyl groups respectively. These VAS scores were statistically comparable in the two groups (Table 3). The time to first dose of analgesic was significantly less (43.70 ± 51.145 min) in Sufentanil group as compared to Fentanyl group (70.68 ± 65.538 min), however the total number of doses needed till the next day morning were (1.60 ± 0.571) in Sufentanil group and (1.44 ± 0.611) which were comparable statistically (Table 4).

**Table 3:** Statistical analysis and comparison in mean of VAS on Extubation and VAS on next morning between the two groups

	Group	Mean	Std. Deviation	p'value (‘t’ test)
VAS on	sufentanil	0.54	1.417	0.390
Extubation	fentanyl	0.32	1.115	
VAS on	sufentanil	0.46	.734	0.772
next morning or after 12 hrs	fentanyl	0.42	.642	

**Table 4:** Statistical analysis and comparison of time (min) for first dose of Tramadol and number of Tramadol doses between the two groups

	Group	Mean	Std. Deviation	p'value (‘t’ test)
Time of 1 st dose of tramadol(min)	sufentanil	43.70	51.145	0.025
	fentanyl	70.68	65.538	
Number of tramadol doses	sufentanil	1.60	0.571	0.180
	fentanyl	1.44	0.611	

Thirty–two out of fifty patients (64%) from the Sufentanil group could be extubated on table while from Fentanyl group, 19 patients (38%) could be extubated on table. Out of the 18 patients from Sufentanil group needing mechanical ventilation, the indication was deep sedation in 13 patients and heavy inotropic supportin 5 patients. Out of the 3l patients from Fentanyl group needing mechanical ventilation, the indication was deep sedation in 20 patients and heavy inotropic support in 11 patients. The mode of ventilation in all patients was SIMV + PSV (10 cmH2O) + CPAP (3-5 cmH2O) with TV 10 ml/kg & RR 10 breaths /min. The average time on mechanical ventilation was 63.10 +99.994 minutes (Range-60-360 minutes) in Sufentanil group (36% patients) and 119.90 + 126.996 minutes (Range-25-360 minutes) in Fentanyl group (62% patients). None of the patients from either groups required mechanical ventilation exceeding 6 hours.

This difference in the number of patients needing mechanical ventilation and the ventilation time was statistically significant. However, this difference in ventilation time did not affect the duration of ICU stay which was 1.16 + 0.370 days in Sufentanil group and 1.14 + 0.351 days in Fentanyl group which was comparable (Tables 5‐6).

**Table 5:** Statistical analysis and comparison of number of Patients needing Mechanical ventilation b/w 2 groups

		Group		Total		p'value
		sufentanil	fentanyl			(Pearson Chi-Square)
	NO	n	32	19	51	
mechanical ventilation		% within group	64.0%	38.0%	51.0%	
	YES	n	18	31	49	**0.009**
		% within group	36.0%	62.0%	49.0%	
		n	50	50	100	
		% within group	100.0%	100.0%	100.0%	

**Table 6:** Statistical analysis and comparison of mean of Total time (min) of mechanical ventilation between the two groups.

	group	N	Mean		Std. Deviation	p'value (‘t’ test)
Mechanical	sufentanil	50	63.10		99.994	.015
ventilation time (min)	fentanyl	50	119.90		126.996	
ICU Stay (Days)	sufentanil	1.16		.370		0.782
	fentanyl	1.14		.351	

Only one patient out of hundred belonging to Sufentanil group had awareness in the form of explicit memory of sound of sternotomy which lasted for few seconds. This patient was referred for psychological counseling.

None of the patients from either group had vomiting in postoperative period. One patient from Fentanyl group complained of mild nausea. This low incidence of PONV could have been because:-

- All patients were given antiemetic prophylax is prior to extubation in the form of Inj. Ondansetron O.8mg/kg.
- We did not use very high doses of opioids.
- Local anaesthetic infiltration of the surgical site reduced requirement of Inj. Tramadol

## Discussion

Conventional practice of cardiac anaesthesia included high dose of opioid agents and prolonged post operative elective mechanical ventilation which in turnled to prolonged ICU stay and a protracted recovery. With the advent in surgical technique, warm by pass and anaesthesia; “Fast–Tracking” has become a reality Fast–Tracking incorporates early extubation leading to early mobilization and rehabilitation of patients^9^. Early extubation improves cardiac performance due to increased ventricular filling and reduces the incidence of postoperative pulmonary complications such as atelectesis.^9^ Early mobilization has also been shown to improve patients’ emotional well being. Fast tracking also shortens ICU and effectively hospital stay resulting in reduction of cost and better resource utilization^5^. A growing body of evidence from randomized trials has identified many anesthetic interventions in Fast Track cardiac anaesthesia (FTCA) that can improve outcome after cardiac surgery^10^. These include new short-acting hypnotic, opioid, and neuromuscular blockingdrugs. Fentanyl, Sufentanil, Remifentanil have been used effectively for FTCA in many studies.^4^‐^7^ Sufentanil is 5-10 times more potent than Fentanyl and has a shorter duration of action than Fentanyl. We hypothesized that Sufentanil would shorten the time for extubation and ICU stay.

The purpose of this study was to compare the effects of two different opioid drugs Fentanyl and Sufentanil for cardiac surgery with respect to time to extubation, postoperative pain, hemodynamic stability and time to intensive care unit discharge and awareness during surgery. This was a prospective, randomized double blind study of 100 patients, 50 in each group labeled as GROUP ‘S’ and GROUP ‘F’ (another two patients were excluded from study because of prolonged ventilation >6 hrs secondary to surgical complication).

3μg.kg^-1^ of fentanyl was used during induction in ‘F’ group. As sufentanil is 5-10 times more potent than Fentanyl with half the duration of action, loading dose of 0.5μgkg^-1^ of sufentanil was used for induction in ‘S’ group. For subsequent doses, lμg.kg^-1^ of fentanyl and 0.lμg.kg^-1^ of sufentanil was given respectively and the total dose fentanyl and sufentanil was restricted to 6μg.kg^-1^ and μg.kg^-1^ respectively. We used low doses offentanyl and sufentanil compared to most studies in literature as the patient population comingto our setup with valvular or congenital heart disease belongs to low socio-economic society with poor general condition and this dose range was found to be adequate in pilot cases.

There was no statistically significant difference in the two groups with respect to age, weight, sex and in percent distribution of diagnos is among the two groups. Thus both the groups were comparable with respect to demographic parameters and surgical diagnosis. There was no statistically significant difference in the preoperative hemodynamic parameters making the two groups comparable in terms of baseline parameters.

Throughout the prebypass and postbypass period hemo dynamic parameters, SpO_2_ and temperature were monitored every 5 min. There was no statistically significant difference in these parameters in both the groups throughout prebypass and postbypass period. There was no statistically significant difference between two groups in the number of patients needing ionotropic support as well as the dose of ionotropic agent. Thus both Sufentanil and Fentanyl provide good hemodynamic stability.

More number of patients in Sufentanil group (32 i.e. 64%) could be extubated on table and did not need mechanical ventilation as compared to 19 patients (i.e.38%) in Fentanylgroup. The mean time of mechanical ventilation was 63.10 min in Sufentanil group as compared to 119.90 min in Fentanyl group. Thus, time for mechanical ventilation in Sufentanil group was found to be reduced than that in Fentanyl group by an average of 57 minutes. This difference in the number of patients needing mechanical ventilation and duration of mechanical ventilation was statistically significant, indicating that extubation is achieved earlier in Sufentanil group. However, this difference did not affect the length of ICU stay which was comparable in both groups.

Our results are similar to those of Butterworth, John MD; James, Robert Stat et al^11^ who found that use of Sufentanil rather than Fentanyl was associated with a significant (*p* =0.045) reduction (of 1.9 h 95% CI, 0.04 to 4.1 h) in duration of time to extubation and no significant effect on ICU length of stay after extubation.

London MJ,^12^ did recent observational study of Butterworth et alusing “mixed-effects” logistic regression modeling of a 40 hospital “benchmarking” data-set, and found little effect of use of Sufentanil (over Fentanyl) on ICU or total length of stay (after adjustment for patient risk and hospital level effects) but, Sufentanil use was associated with a 1.9 hr. reduction in time to extubation. Our results are similar.

In our study, no statistically significant difference was found between two groups with regards the VAS score after extubation and VAS on the next day morning or after 12 hours. In terms of the time for first dose of Tramadol required, it was found to be earlier with Sufentanil than Fentanyl 43.70 ± 51.145 min vs.70.68 ± 65.538 min; which is statistically significant. This is obvious as Fentanyl has longer duration of action compared to Sufentanil. But the total doses of Tramadol required in the postoperative period till next morning were similar. Engoren et al^13^ studied patients undergoing cardiac surgery which were randomized to a Fentanyl-based, Sufentanil-based, or remifentanil-based anesthetic. Postoperative pain was measured at 30 min after extubation and at 6:30 AM on the first postoperative day. Pain scores at both times were similar in allthree groups (P>0.05).

Cardiac anaesthesia is associated with higher incidence of awareness compared with other specialties. The incidence reported ranges from 1.1% to 23% depending upon the dose and agents used in anaesthesia. Possible reasons for this are, use of high opioid based techniques which reduces requirement of inhalational and intravenous anesthetic agents, almost unpredictable phamnacodynamics of anaesthetics under the extracorporeal circulation especially in the rewarnning period and at the time of cessation of bypass, interpersonal and interracial differences in drug reactions, haemodilution, and binding on foreign surface areas.

Dowd et al^14^ did aprospective study on incidence of awareness in cardiac anaesthesia and reported an incidence of 0.3% in fast-track cardiac anaesthesia. This low incidence of awareness was related to the use of a balanced anesthetic technique involving the continuous administration of volatile (isoflurane) or intravenous (propofol) anaesthetic agents before, during, and after cardiopulmonary bypass. We too, used a balanced anesthetic technique.

In our study, one case from Sufentanil group had awareness during anaesthesia. The overall incidence of awareness in our study was 1.7% which was statistically insignificant. We did not use a BIS monitor and incidence of awareness in our study could have been probably avoided using BIS monitor.

In conclusion, both sufentanil and fentanyl provide hemodynamic stability, early recovery and equal VAS scores in postoperative period, though fentanyl provides longer duration of postoperative analgesia. Sufentanil allows earlier extubation but duration of ICU stay is similar with both drugs. There is no statistically significant difference in incidence of awareness in the two groups. Thus, boththe agents can be used for Fast-Track cardiac anaesthesia (FTCA), effectively. A use of BIS monitor is advisable to prevent awareness.
